# Long-term cryostorage does not impair human sperm motility: evidence from a three-decade cohort study

**DOI:** 10.1007/s10815-026-03962-7

**Published:** 2026-07-07

**Authors:** Shimi Barda, Shanee Dim, Shlomit Shabat, Sandra Edith Kleiman, Noga Fuchs Weizman

**Affiliations:** 1 Institute for the Study of Fertility, Lis Maternity Hospital, Tel Aviv Sourasky Medical Center, Tel Aviv, Israel; 2 Gray Faculty of Medical and Health Sciences, Tel Aviv University, Tel Aviv, Israel; 3 Israel Academic College, Ramat Gan, Israel

**Keywords:** Sperm cryopreservation, Long-term cryostorage, Sperm motility, Post-thaw quality, Cryoinjury, Sperm banking

## Abstract

**Purpose:**

To evaluate whether long-term cryostorage duration affects post-thaw sperm motility and progressive motile concentration (PMC) in samples cryopreserved for up to 30 years.

**Methods:**

A retrospective cohort study of 629 donor sperm samples cryopreserved between 1990 and 2020 in a single university-affiliated andrology laboratory was conducted. Motility and PMC were assessed immediately after thawing (T0) and after extended storage (TES). Associations between storage duration and post-thaw changes were evaluated using correlation analysis, multivariable linear regression, and multilevel mixed-effects models with donor-level random effects. K-means clustering was applied to identify sperm quality phenotypes.

**Results:**

Total motility declined significantly following the freeze–thaw process (*p* < 0.001) but remained within clinically usable ranges after extended storage. Storage duration showed only a weak association with relative motility change (*r* = 0.169, *p* < 0.001), without a consistent duration-dependent trend in absolute post-thaw motility. PMC changes were modest and non-monotonic across storage groups. Multilevel models indicated that storage duration explained only a small proportion of variance in long-term outcomes. Cluster analysis identified distinct sperm quality phenotypes independent of storage duration.

**Conclusions:**

Long-term cryostorage of up to 30 years is not associated with substantial impairment of sperm motility or PMC. These findings support the stability of cryopreserved spermatozoa under prolonged storage conditions.

## Introduction

Cryopreservation of human spermatozoa is a cornerstone of assisted reproductive technology, enabling donor insemination and fertility preservation before gonadotoxic therapies or gender-affirming treatments, and allowing flexibility in timing between gamete collection and clinical use [1]. Although the technique has been used for decades, uncertainty persists regarding the long-term stability of sperm quality during extended cryostorage periods.

It is well established that freeze–thaw cycles inflict severe cellular stress and damage through multiple mechanisms: ice crystal formation causes mechanical damage to membranes, rapid osmotic shifts during cryoprotectant addition and removal stress cellular structures, and oxidative damage accumulates from reactive oxygen species [2]. During the initial freezing, spermatozoa experience temperature transitions that trigger phase changes in membrane lipids, potentially compromising membrane integrity and mitochondrial function. While storage at − 196 °C effectively halts biological processes, questions remain about whether subtle degradation continues to occur over time [3].

Previous investigations into the effects of storage duration have yielded inconsistent findings, creating uncertainty for clinicians and patients. Some studies suggest a gradual, time-dependent decline in post-thaw sperm quality, particularly in motility and viability, accompanied by increased oxidative stress and DNA fragmentation with prolonged storage [4, 5]. In contrast, other reports indicate that although laboratory parameters may show modest deterioration over time [6], long-term cryostorage does not adversely affect clinical outcomes, with pregnancy and live birth rates remaining stable even after many years of storage [7]. These inconsistencies likely stem from methodological limitations in prior studies, including small sample sizes, inconsistent cryopreservation protocols, and inadequate control for baseline sample quality or donor-specific biological variability. Moreover, procedural variables such as transfer between containers, access frequency, and thermal fluctuations during handling have rarely been documented or considered as potential confounders in previous studies.

The clinical implications of resolving this controversy are substantial. Young cancer patients must decide whether to bank sperm before treatment, investing financially and emotionally in samples that may not be used for decades. Sperm banks face decisions about storage duration limits, disposal policies, and quality assurance protocols. Regulatory bodies require evidence-based guidelines for storage regulations. Most importantly, patients deserve accurate information about the longevity of their preserved fertility.

To address the methodological limitations of previous studies, we analyzed a large, systematically archived cohort of sperm samples cryopreserved for up to 30 years at a single university-affiliated andrology laboratory. A key strength of this study is the long-term consistency of laboratory protocols, enabling evaluation of storage effects under stable conditions. Using a comprehensive analytical strategy, we examined not only whether storage duration is associated with post-thaw sperm motility, but also the direction and determinants of this relationship. Specifically, we sought to distinguish between true storage-duration-dependent deterioration and patterns arising from intrinsic characteristics of the sample related to cryosurvival at the time of freezing. Examining the direction of motility change therefore provides a critical framework for determining whether long-term post-thaw outcomes reflect prolonged storage itself or properties intrinsic to the sample.

## Materials and methods

### Ethics approval

This study was approved by the Institutional Review Board of a tertiary university-affiliated medical center (IRB No. 0401–24). The requirement for informed consent for the present study was waived due to its retrospective design and the use of de-identified data. All procedures were conducted in accordance with the principles of the Declaration of Helsinki.

### Study design, population, and selection criteria

This retrospective cohort study examined cryopreserved sperm samples from young adult males who donated sperm at a tertiary university-affiliated medical center sperm bank between 1990 and 2020. All donors met national eligibility criteria, including normal physical examination findings, absence of genetic disorders, and negative screening for sexually transmitted infections. At the time of donation, donors provided written consent for the clinical use of their samples and for anonymized research use in accordance with institutional policy.

Samples were categorized into four predefined storage-duration groups: 5–10, 10–15, 15–20, and > 20 years. Storage duration was analyzed both as a continuous variable and as predefined categorical intervals to allow evaluation of potential duration-related trends across storage periods. Semen parameters were recorded at three time points for each sample: (1) fresh, prior to cryopreservation, (2) immediately after thawing for initial post-freeze quality control (T0), and (3) after extended storage when thawed for this study (TES). The primary comparison was between T0 and TES, enabling assessment of whether extended storage independently affects post-thaw sperm quality. Primary outcome measures included overall post-thaw motility percentage and post-thaw progressive motility concentration (PMC). Secondary outcomes included post-thaw motility grades according to WHO classification and clustering patterns based on immediate post-thaw sperm quality parameters.

We included samples with complete documentation of both cryopreservation and thawing processes, including pre-freeze and post-thaw quality parameters and precise cryostorage duration. We excluded samples that lacked essential data (e.g., thawing analysis or storage duration); were intended for purposes other than donation (e.g., fertility preservation in cancer patients); or showed compromise during storage or thawing (e.g., mechanical damage, labeling errors).

### Sample collection and processing

All procedures related to sperm collection, processing, and cryopreservation remained consistent throughout the study period (1990–2020). All procedures were conducted in a certified andrology laboratory by experienced laboratory technicians. Semen samples were collected via masturbation into sterile, pre-labeled containers in a private collection room within the sperm bank facility. Donors maintained 2–3 days of sexual abstinence prior to collection.

### Semen analysis and motility assessment

Following collection, each ejaculate was allowed to liquefy at 37 °C for a minimum of 30 min before analysis. Sperm quality was assessed prior to cryopreservation. Semen analysis included measurement of volume, sperm concentration, motility, and morphology. Concentration was determined using a Makler counting chamber. Total motility percentage was evaluated at × 400 magnification across at least five microscopic fields, with a minimum of 200 spermatozoa counted. Morphological assessment was performed on Papanicolaou-stained slides under oil immersion at × 1000 magnification, following the strict criteria described by Menkveld and Kruger [8] and the WHO laboratory manual 5th edition [9]. Morphology data were recorded as part of routine semen analysis but were not included in the present analysis, as the study focused on motility-based outcomes and progressive motile concentration as the primary measures of post-thaw sperm function.

Because the study spans three decades (1990–2020), semen motility assessments were originally performed according to the WHO laboratory manual editions that were in use at the time of analysis. Earlier samples were evaluated according to WHO 1999 criteria [10], whereas later assessments followed WHO 2010 guidelines [9]. Although the classification of progressive motility differs slightly between these editions, total motility (progressive and non-progressive spermatozoa) is defined consistently across both systems. To ensure methodological comparability across the cohort, total motility percentage was therefore used as the primary parameter for cross-timepoint motility analyses.

### Cryopreservation protocol

Samples were processed to achieve a target post-thaw progressive motile sperm concentration of 8–12 × 10⁶/mL. Prior to cryopreservation, each sample underwent a standardized washing procedure. The liquefied semen was diluted 1:1 with modified human tubal fluid (mHTF; Irvine Scientific, Santa Ana, CA, USA) supplemented with 1% human serum albumin (Bio Products Laboratory, UK), followed by centrifugation at 300 g for 10 min. The supernatant was discarded, and the pellet was resuspended in fresh mHTF medium.

Cryoprotective medium containing egg yolk buffer (Irvine Scientific, USA) was added gradually to the semen sample to minimize osmotic stress. After reaching a final 1:1 dilution of semen and cryoprotectant, the mixture was equilibrated for 20 min at room temperature and loaded into 0.5 mL high-security straws. The number of straws prepared per ejaculate was recorded and varied according to specimen volume and concentration, with each straw constituting a single therapeutic insemination dose. One designated straw per sample was allocated for immediate post-thaw quality control (T0). Samples were frozen using a controlled-rate slow-freezing protocol with a cooling rate of − 1 °C/min until − 80 °C, then stored in liquid nitrogen at − 196 °C.

### Storage and handling practices

All samples were continuously stored in liquid nitrogen at − 196 °C in dedicated cryogenic storage tanks within the sperm bank facility. Samples designated for clinical donor programs underwent regular inventory assessments and periodic transfers between storage containers as part of routine operational procedures. Access frequency varied according to donor demand and clinical utilization. Once samples were reclassified for long-term research use (i.e., after donors had reached the maximal permitted number of family units), they were segregated into dedicated storage tanks with minimal disturbance. These samples remained in their original storage location with substantially reduced handling frequency and were accessed only for periodic quality monitoring or at predefined study endpoints.

### Thawing and post-thaw analysis

For thawing, straws were placed on a 37 °C heating plate for 10 min. The thawed contents were then transferred into a sterile test tube and gently mixed using a vortex to ensure homogeneity.

Initial post-freeze quality control straws (T0) were thawed immediately after cryopreservation and assessed using manual microscopic evaluation at × 400 magnification for overall motility percentage and progressive motile sperm concentration, following the same methodology as pre-freeze analysis.

Long-term storage samples (thawed after extended storage; TES) were assessed using a computer-assisted sperm analysis (CASA) system (Microptic, Spain). The CASA system digitally captured at least six microscopic fields per sample and automatically quantified sperm concentration and motility. Motility was classified according to WHO grades: A (rapid progressive), B (slow or sluggish progressive), C (non-progressive), and D (immotile), reflecting the quality of sperm motility.

To address potential measurement differences between manual microscopy and CASA analysis, a validation study was performed on a subset of samples (*n* = 50) analyzed using both methods. The results demonstrated strong correlation between techniques (r = 0.94, *p* < 0.001), with small mean differences (< 3% for motility and < 5% for PMC), indicating good measurement consistency between methods.

### Quality control measures

Internal quality assurance included regular instrument calibration, periodic proficiency testing, and participation in external quality assessment programs, including the external quality assessment service (NEQAS), with quarterly evaluations. All measurements were performed in duplicate to ensure data consistency. Additional recorded data included the identity of the laboratory technician performing each analysis, the exact dates of freezing and thawing, and the number of straws prepared per sample.

### Statistical analysis

Statistical analyses were conducted using IBM SPSS version 18.0, JMP (SAS Institute), and R version 4.4.1. A two-sided alpha level of 0.05 was considered statistically significant. Quantitative variables were analyzed using paired-sample t-tests for within-subject differences and one-way ANOVA with Tukey–Kramer HSD post hoc tests for comparing storage duration groups. Linear correlation analyses examined associations between storage duration and sperm quality outcomes. For multivariate relationships, appropriate regression models were applied depending on variable structure.

Advanced methods included K-means clustering to identify post-thaw sperm quality patterns independent of storage duration. The optimal number of clusters (*k* = 2) was determined using the elbow method and silhouette analysis. Multilevel modeling (MLM) was employed to account for hierarchical data structure where multiple samples originated from the same donor, and to control for intra-individual variability. Generalized linear models (GLM) were used to explore combined predictor effects on post-thaw outcomes. Results were analyzed both by group and across all donor observations to enable robust assessment of intra-donor stability over time. As part of statistical quality control, potential technician-related effects on sperm parameters were evaluated.

## Results

### Study population and sample characteristics

A total of 629 cryopreserved donor samples met inclusion criteria for analysis. Storage duration ranged from 5.6 to 30.5 years (mean 16.5 ± 5.8 years). Samples were distributed across four storage-duration groups: 5–10 years (*n* = 159), 10–15 years (*n* = 137), 15–20 years (*n* = 185), and > 20 years (*n* = 148). The final dataset comprised samples from 52 donors, with a median of 8 samples per donor (IQR 3–16; range 1–39). Donor age at the time of cryopreservation ranged from 20 to 31 years (mean 24.6 ± 5.2 years).

Baseline sample characteristics are summarized in Table 1. Fresh total motility percentage differed significantly across storage groups (ANOVA *p* < 0.0001), with values ranging from 58.71 ± 6.23% in the > 20-year group to 67.28 ± 4.76% in the 5–10 year group.

**Table 1:** Sperm quality parameters by cryostorage duration group. Values are presented as mean (± SD)

Parameter	5–10 (*n* = 159)	10–15 (*n* = 137)	15–20 (n = 185)	> 20 (*n* = 148)	Overall (*N* = 629)	*p*-value
**Motility parameters**						
Fresh total motility (%)	67.28 (± 4.76)	64.86 (± 6.35)	61.97 (± 5.12)	58.71 (± 6.23)	63.17 (± 6.42)	** <.0001**
T0 total motility (%)	50.11 (± 4.65)	48.34 (± 6.11)	48.43 (± 4.77)	41.66 (± 7.94)	47.24 (± 6.71)	** <.0001**
TES total motility (%)	36.77 (± 7.41)	38.30 (± 7.22)	38.20 (± 8.99)	33.75 (± 8.77)	36.81 (± 8.37)	** <.0001**
Δ Motility, % (T0 → TES) *	− 26.28 (± 14.65)	− 20.21 (± 14.24)	− 21.22 (± 16.35)	− 18.48 (± 16.27)	− 21.63 (± 15.70)	** <.0001**
**TES motility grade distribution**						
Grade A (%)	5.56 (± 2.53)	5.59 (± 3.09)	5.27 (± 3.00)	4.88 (± 3.18)	5.32 (± 2.96)	0.145
Grade B (%)	14.36 (± 3.72)	14.96 (± 4.44)	15.61 (± 4.78)	12.81 (± 4.89)	14.49 (± 4.60)	** <.0001**
Grade C (%)	16.85 (± 3.82)	17.79 (± 4.62)	17.32 (± 4.46)	16.07 (± 3.98)	17.01 (± 4.27)	**0.005**
Grade D (%)	63.23 (± 7.41)	61.70 (± 7.22)	61.80 (± 8.99)	66.25 (± 8.77)	63.19 (± 8.37)	** <.0001**
**Progressive motile concentration**						
T0 PMC (× 10⁶/mL)	10.00 (± 2.47)	11.08 (± 3.06)	11.68 (± 3.21)	10.54 (± 4.30)	10.86 (± 3.37)	** <.0001**
TES PMC (× 10⁶/mL)	9.94 (± 4.95)	15.52 (± 6.78)	15.59 (± 8.38)	10.12 (± 5.09)	12.86 (± 7.10)	** <.0001**
Δlog PMC (%)	− 3.67 (± 24.39)	12.53 (± 18.98)	7.13 (± 21.39)	− 3.55 (± 19.04)	3.06 (± 22.20)	** <.0001**

^*^Relative change calculated as (TES − T0)/T0 × 100

Motility grades: *A* rapid progressive, *B* slow progressive, *C* non-progressive, *D* immotile

Abbreviations: *T0* immediate post-thaw assessment, *TES* thaw after extended storage, *PMC *progressive motile concentration, *Δ* change

*p*-values from one-way ANOVA. Bold *p*-values indicate statistical significance (*p* < 0.05)

### Impact of freeze–thaw on immediate and long-term sperm motility

All storage-duration groups demonstrated a significant decline in total motility from fresh assessment to immediate post-thaw assessment (T0) (paired *t*-test, *p* < 0.001 for all groups, Table 1).

A further decline was observed following extended storage (TES), with reduced motility and an increased proportion of low-grade motility across all groups.

While the magnitude of decline varied significantly between groups, final post-thaw values were clinically comparable. All groups maintained clinically usable sperm parameters (Table 1). TES motility values ranged from 33.75 ± 8.77% in the > 20-year group to 38.30 ± 7.22% in the 10–15-year group. The mean overall relative reduction in motility across all samples was 21.63%.

### Relationship between storage duration and post-thaw motility change

**Fig. 1:**
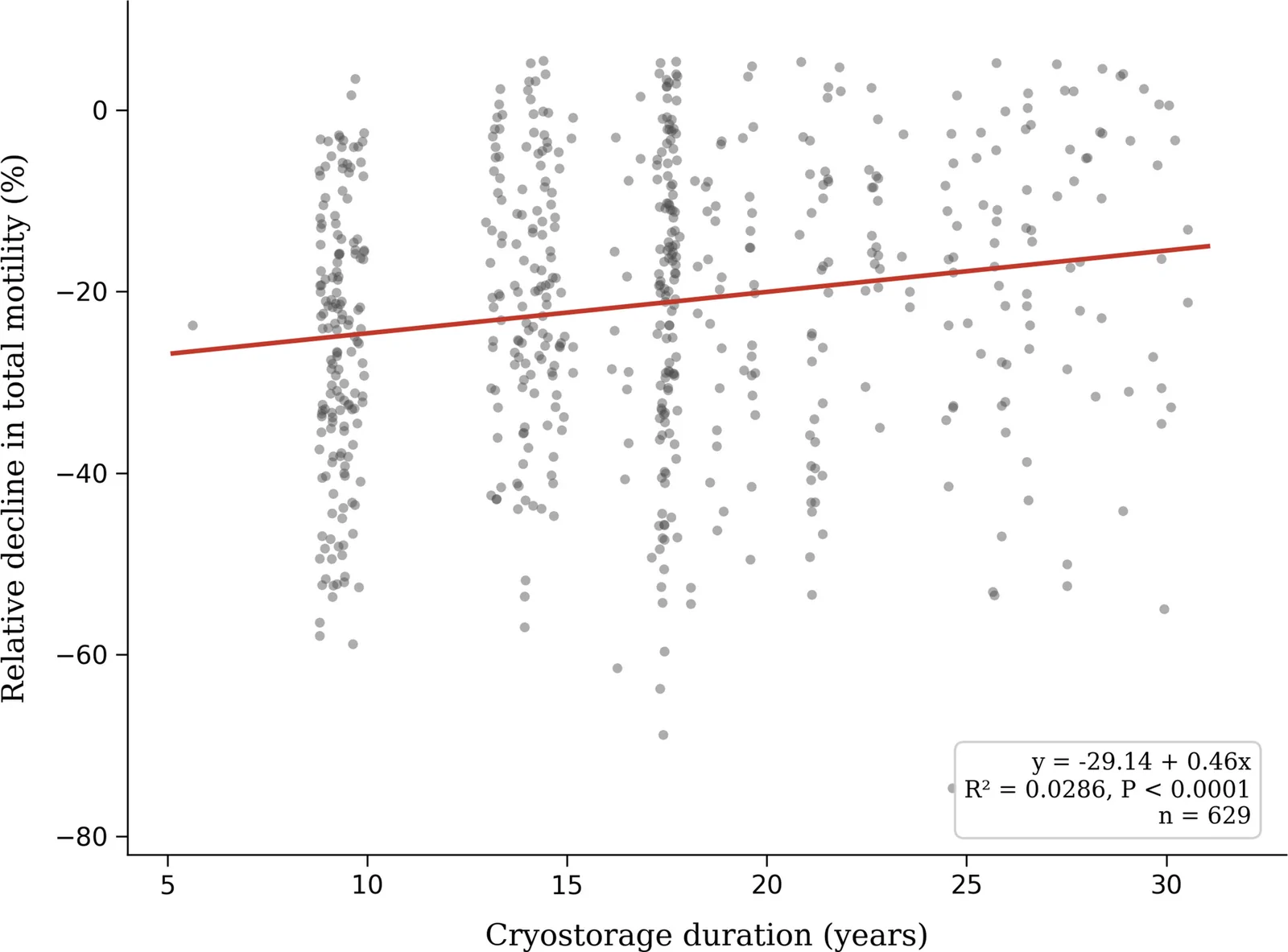
Association between cryostorage duration and relative decline in post-thaw sperm motility. Scatter plot showing the relationship between sperm cryostorage duration (years) and the relative decline in total motility between the initial post-thaw assessment (T0) and the post-thaw assessment after extended storage (TES). Each point represents one thawed sample (*n* = 629). The red line represents the linear regression fit, demonstrating a weak but statistically significant association between storage duration and the relative decline in motility (*R*^2^ = 0.0286, *p* < 0.0001)

Mean relative Δ motility [(TES − T0)/T0 × 100] values were − 26.28% (5–10 years), − 20.21% (10–15 years), − 21.22% (15–20 years), and − 18.48% (> 20 years). A weak positive correlation was observed between storage duration and Δ relative motility (Pearson *r* = 0.169, *p* < 0.001, Fig. 1), indicating that the magnitude of motility decline from T0 to TES tended to be smaller in longer-stored samples. This direction of association, lesser decline with longer storage duration, was accompanied by a systematic difference in baseline T0 motility across cohorts, with the longest-stored group exhibiting the lowest T0 values (41.66 ± 7.94%) and the shortest-stored group the highest (50.11 ± 4.65%; Table 1). Notably, the group demonstrating the greatest relative motility decline was also the group with the highest initial post-thaw quality rather than the longest storage duration.

### Progressive motility concentration

The overall PMC at T0 was 10.86 (± 3.37) × 10⁶/mL and at TES was 12.86 (± 7.10) × 10⁶/mL. Raw PMC change (ΔPMC: 19.03 ± 55.09%) exhibited substantial variability due to right-skewed distribution; analyses were therefore performed on log-transformed data.

On the log scale, overall change between T0 and TES was small (log PMC: T0 = 1.01 ± 0.15; TES = 1.04 ± 0.26; Δlog PMC = 3.06 ± 22.20%). Log PMC changes showed significant between-group variation (one-way ANOVA, *p* < 0.0001) but did not follow a consistent trend across storage-duration groups. Δlog PMC was − 3.67% (± 24.39) in the 5–10-year group, + 12.53% (± 18.98) in the 10–15-year group, + 7.13% (± 21.39) in the 15–20-year group, and − 3.55% (± 19.04) in the > 20-year group (Table 1). The shortest and longest storage groups exhibited minimal change, whereas the intermediate groups showed larger positive changes (*p* < 0.001 for both).

### Motility grade distributions

TES motility grade distributions were broadly similar across storage-duration groups and showed no consistent duration-dependent pattern (Table 1). Overall mean proportions were 5.32% rapid progressive (grade A), 14.49% slow progressive (grade B), 17.01% non-progressive (grade C), and 63.19% immotile (grade D). Storage duration showed weak negative correlations with long-term Grade A and Grade B motility, with no significant association for Grade C (Pearson *r* =  − 0.102, − 0.130, and − 0.068, respectively; *p* = 0.0105, 0.0010, and 0.090). Effect sizes were small (*R*^2^ ≤ 0.017), indicating minimal variation in motile grade distribution across storage durations.

### Predictors of post-thaw motility

To identify factors predicting the magnitude of motility change during extended storage, we constructed a generalized linear model (GLM) with relative Δ motility [(TES − T0)/T0 × 100] as the dependent variable and pre-freeze motility (fresh), immediate post-thaw motility (T0), and storage duration as independent predictors. The model explained a small proportion of the variance (*R*^2^ = 0.051; adjusted *R*^2^ = 0.047). Individual predictor estimates were as follows: fresh pre-freeze motility (*β* = 0.183, *p* = 0.118), T0 (*β* =  − 0.409, *p* = 0.0001), and storage duration (*β* = 0.348, *p* = 0.0074).

### Multilevel modeling of post-thaw sperm quality

Multilevel mixed-effects models were fitted with donor identity specified as a random intercept to account for repeated sampling. In the model predicting relative change in total motility between T0 and TES, immediate post-thaw motility (T0) and storage duration were significant independent predictors, whereas fresh pre-freeze motility was not (T0: *β* =  − 0.322, *p* = 0.0064; storage duration: *β* = 0.564, *p* = 0.0349; fresh pre-freeze: *β* = 0.144, *p* = 0.2707). The coefficient for T0 was negative, indicating that higher immediate post-thaw motility was associated with greater relative motility decline during extended storage. The coefficient for storage duration was positive, indicating that longer storage duration was associated with smaller relative motility decline. The donor-level random intercept accounted for 19.3% of total variance (intraclass correlation coefficient (ICC) = 0.193).

In the model predicting change in PMC between T0 and TES, storage duration showed a small negative association (*β* =  − 2.09 × 10⁶/mL per year, *p* = 0.0252), whereas the donor-level random effect accounted for 25.3% of total variance (ICC = 0.253).

### Cluster analysis of sperm quality phenotypes

**Fig. 2:**
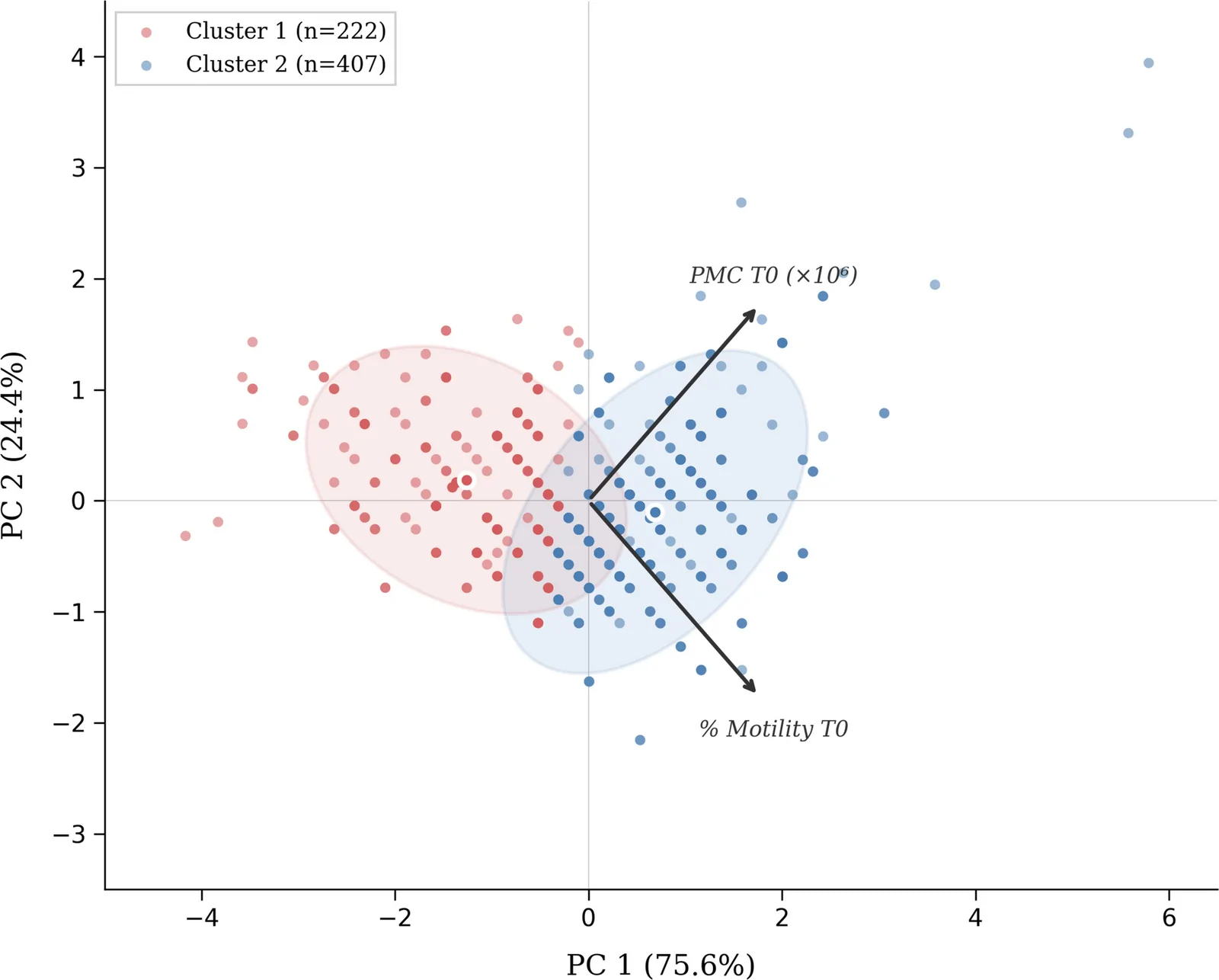
Cluster analysis identifying two distinct populations based on immediate post-thaw sperm quality. Principal component biplot showing K-means clustering (k = 2) of thawed sperm samples based on post-thaw motility and progressive motile concentration (PMC) at T0. PC1 and PC2 account for 75.6% and 24.4% of the total variance, respectively. Two distinct populations were identified corresponding to lower (Cluster 1, *n* = 222) versus higher (Cluster 2, *n* = 407) post-thaw sperm quality. Ellipses represent 95% confidence regions for each cluster

To assess whether samples could be categorized into distinct quality groups independent of storage duration, unsupervised K-means clustering was performed using immediate post-thaw motility (T0) and PMC. Two distinct sperm-quality phenotypes were identified (Fig. 2). Cluster 1 (*n* = 222) comprised lower-quality samples with reduced T0 motility and PMC (mean T0 motility 40.4%; PMC 8.30 × 10⁶/mL), whereas Cluster 2 (*n* = 407) comprised higher-quality samples (mean T0 motility 51.0%; PMC 12.25 × 10⁶/mL). These clusters therefore reflect intrinsic differences in post-thaw sperm quality rather than the duration of cryostorage. Cluster 1, which comprised lower-quality samples, also had a longer mean storage duration (18.7 years) compared with Cluster 2 (15.3 years), yet demonstrated no greater motility decline during extended storage.

In mixed-effects models fitted within each cluster, immediate post-thaw motility (T0) remained the strongest predictor of TES motility change (Cluster 1: *β* =  − 0.460, *p* = 0.0487; Cluster 2: *β* =  − 0.688, *p* = 0.0042), whereas storage duration was not significant in either cluster (Cluster 1: *β* = 0.517, *p* = 0.1379; Cluster 2: *β* = 0.383, *p* = 0.2693). The donor-level random effect accounted for substantial variability in both clusters (ICC = 0.255 and 0.228, respectively).

## Discussion

This retrospective cohort study analyzed 629 semen samples cryopreserved over three decades under consistent laboratory protocols to determine whether storage duration independently affects sperm quality. The primary objective was to disentangle the effects of storage duration from intrinsic sample characteristics using complementary statistical and multilevel modeling approaches while accounting for intra- and inter-donor variability.

Samples stored for > 20 years exhibited lower pre-freeze motility than more recently cryopreserved samples, indicating temporal variation in baseline semen quality across storage-duration cohorts. Although population-based studies have reported declining semen parameters over recent decades [11–13], the opposite pattern observed here likely reflects the highly selected nature of sperm donor populations and possible evolution of donor recruitment and screening practices over time. Thus, cohort effects related to donor selection should be considered when interpreting comparisons across storage groups.

Immediate post-thaw motility (T0) consistently emerged as the strongest predictor of TES outcomes, whereas pre-freeze motility did not. Although higher initial post-thaw motility (T0) was associated with greater relative decline, this relationship likely reflects statistical and scaling effects inherent to relative change measures, as well as potential floor constraints, rather than a true duration-dependent biological process. Therefore, emphasis was placed on absolute post-thaw outcomes rather than relative change metrics. Importantly, this observation does not support a duration-dependent degradation model, as absolute post-thaw motility values remained comparable across storage durations.

Taken together, these findings are consistent with a front-loaded cryoinjury framework, in which the majority of functional impairment occurs during the freeze–thaw process rather than during prolonged storage. These observations have direct clinical implications. For patients undergoing gonadotoxic treatments, the findings provide reassurance that appropriately cryopreserved samples can maintain post-thaw motility parameters compatible with clinical use after extended storage periods [14–16].

### The role of handling practices in long-term cryostorage

An additional factor potentially influencing long-term post-thaw outcomes is the differential handling history of samples across storage-duration groups. Samples stored for shorter durations remained in active clinical use for a larger proportion of their storage period and therefore underwent more frequent inventory assessments and container transfers, whereas samples transitioned to long-term storage experienced minimal disturbance after segregation into dedicated tanks.

This handling pattern is inversely related to storage duration, such that shorter-stored samples were exposed to more repeated manipulations over time. Each transfer event involves transient exposure outside liquid nitrogen and brief thermal perturbation, which may contribute to cumulative sublethal cryoinjury independent of storage time itself. Although individual access events were not quantitatively recorded, the categorical distinction between clinically active and long-term storage phases indicates that handling frequency represents a plausible confounding factor in analyses of long-term cryostorage outcomes. Prospective studies incorporating systematic logging of container access and sample transfers are needed to quantify this effect directly.

### Donor variability: the dominant factor

Multilevel modeling revealed that donor identity accounted for 19–25% of residual variance across models (ICC = 0.193 for motility; ICC = 0.253 for PMC), representing a substantial source of variability. This finding highlights the biological heterogeneity that persists even within rigorously screened donor populations. Inter-individual variability in sperm freezability is well documented: independent of pre-freeze semen quality, some individuals consistently exhibit poorer post-thaw outcomes [17]. This variability likely reflects intrinsic differences in sperm membrane lipid composition, seminal plasma antioxidant capacity, and possibly genetic determinants of cryosurvival [14, 18, 19].

Supporting this observation, our unsupervised cluster analysis identified two distinct sperm quality profiles that were primarily distinguished by immediate post-thaw quality (T0) rather than storage duration. Samples in Cluster 1 exhibited reduced motility profiles and longer average storage duration compared with Cluster 2 (mean storage duration 18.7 vs. 15.3 years), yet within each cluster storage duration was not a significant predictor of TES outcomes, whereas T0 remained significant in both clusters. This reinforces the interpretation that long-term variability in post-thaw sperm quality reflects intrinsic cryosurvival capacity rather than storage duration itself.

### Cryoinjury primarily occurs during the freeze–thaw process

Across all storage groups, motility declined significantly immediately after thawing, with the largest absolute reduction observed in the 5–10 year cohort, despite this group having the shortest storage duration and the highest pre-freeze quality. This pattern is directly inconsistent with a duration-dependent degradation model, which would predict greater decline in longer-stored samples, and instead supports a front-loaded cryoinjury model in which the predominant cellular damage is incurred during freezing and thawing rather than during storage itself. The freeze–thaw cycle imposes substantial cellular stress through ice crystallization, osmotic perturbations, and oxidative injury [4, 20–23], selectively affecting more fragile sperm subpopulations [24–26]. The present findings align with a front-loaded cryoinjury framework, in which the predominant functional impairment occurs during freezing and thawing, while prolonged storage under stable cryogenic conditions contributes minimally to additional decline. Although storage duration reached statistical significance in the multilevel model (*β* = 0.564, *p* = 0.035), the direction of this association indicated smaller relative decline with longer storage, suggesting that the observed relationship likely reflects baseline T0 differences rather than progressive storage-related degradation. These findings are consistent with previous reports demonstrating preserved sperm function after more than two decades of cryostorage [6, 14, 27–30].

### Progressive motility concentration changes

Overall progressive motile concentration (PMC) changed only modestly between T0 and TES (log PMC: 1.01 ± 0.15 vs 1.04 ± 0.26; Δlog PMC = 3.06 ± 22.20%), indicating no meaningful global deterioration during storage. Although log-PMC changes differed significantly between storage-duration groups (ANOVA *p* < 0.0001), they did not follow a consistent duration-dependent trend.

This non-monotonic pattern is unlikely to represent biological improvement during storage and more plausibly reflects differences in baseline cryopreservation conditions between T0 control straws and the originally stored samples. In the present study, T0 measurements were obtained from control straws frozen on the original cryopreservation date and thawed immediately. Differences in specimen volume between T0 control straws and clinical storage straws may also have contributed to the observed PMC pattern. Experimental data demonstrate that specimen volume within identical straw formats influences post-thaw sperm quality, including PMC, with larger volumes yielding improved post-thaw parameters. Accordingly, the modest increases in PMC observed in some groups in our study may partly reflect differences in specimen volume between T0 control straws and stored straws, as straw volume has been shown to influence post-thaw sperm parameters [31]. However, this explanation remains speculative in the absence of direct volume measurements for each straw.

### Clinical significance and practical implications

Although statistically significant differences were detected across storage groups, it is important to distinguish statistical from clinical significance. The motility reductions observed were modest in absolute terms, and all post-thaw motility levels remained within clinically acceptable ranges (TES motility 33.75–38.30%) [18, 32]. Importantly, no consistent duration-dependent decline in absolute post-thaw motility or PMC was observed across storage groups.

For clinical practice, these findings support several practical considerations. First, early post-thaw assessment (T0) appears to be a reliable prognostic indicator for long-term sample viability. Second, optimizing collection conditions and baseline semen quality may be more relevant than imposing arbitrary storage-duration limits. Third, minimizing unnecessary handling and container transfers during storage may reduce potential cumulative cryoinjury.

### Limitations

Several limitations should be considered when interpreting these findings. First, the transition from manual microscopy to CASA-based assessment may introduce measurement variability, despite the strong validation correlation observed between the methods (*r* = 0.94). In addition, quantitative records of sample handling events were not available, and therefore the potential contribution of handling frequency to post-thaw variability remains hypothetical.

Second, the study focused primarily on motility parameters and did not assess other aspects of sperm function, such as DNA integrity, acrosome status, fertilization capacity, embryo development, or live birth outcomes. Although motility is a clinically validated and widely used surrogate marker, it does not fully capture all dimensions of sperm function relevant to reproductive success.

Finally, the cohort consisted exclusively of screened sperm donors who met stringent eligibility criteria at the time of donation, representing a highly selected population with above-average baseline semen parameters. The findings may therefore not be directly generalizable to men banking sperm for medical indications, such as cancer treatment or other gonadotoxic therapies, who may exhibit greater baseline biological vulnerability and different patterns of post-thaw decline.

Future studies integrating molecular markers of cryosurvival [33–35] together with clinical reproductive outcomes would further strengthen the biological and translational interpretation of these findings.

## Conclusion

Our findings indicate that long-term cryostorage is not associated with substantial impairment of human sperm motility. No consistent duration-dependent decline in post-thaw motility or progressive motile concentration was observed across storage periods of up to 30 years. The observed patterns of variability are not consistent with progressive storage-related deterioration and are more likely influenced by intrinsic sample characteristics and methodological factors. These results support the biological stability of cryopreserved spermatozoa under prolonged storage conditions and highlight the clinical relevance of post-thaw assessment in sperm banking practice. Future studies incorporating detailed handling records, clinical reproductive outcomes, and non-donor populations will further clarify the long-term clinical implications of these findings.

## Data Availability

The datasets generated and/or analyzed during the current study are available from the corresponding author on reasonable request.
